# Highly Efficient Symmetric Key Based Authentication and Key Agreement Protocol Using Keccak

**DOI:** 10.3390/s20082160

**Published:** 2020-04-11

**Authors:** An Braeken

**Affiliations:** INDI, Vrije Universiteit Brussel, 1040 Brussel, Belgium; an.braeken@vub.be

**Keywords:** authentication protocol, anonymity, symmetric key based, Keccak

## Abstract

Efficient authentication and key agreement protocols between two entities are required in many application areas. In particular, for client–server type of architectures, the client is mostly represented by a constrained device and thus highly efficient protocols are needed. We propose in this paper two protocols enabling the construction of a mutual authenticated key ensuring anonymity and unlinkability of the client and resisting the most well known attacks. The main difference between the two proposed protocols is in the storage requirements on the server side. The innovation of our protocols relies on the fact that, thanks to the usage of the sponge construction, available in the newly proposed SHA3 standard with underlying Keccak design, the computation cost can be reduced to only one hash operation on the client side in case of the protocol with storage and two hash operations for the protocol without storage and thus leads to a very efficient solution.

## 1. Introduction

There are many devices participating in wireless communications that are constrained in nature, for instance, sensors from wireless body area networks (WBANs), sensors and actuators in smart homes, IoT (Internet of things) devices in Industrial IoT (IIoT), Radio-frequency identification (RFID) tags, smart meters, etc. [1]. In all these settings, in order to ensure secure communication, a common shared key should be first negotiated between the device and a more powerful entity like gateway or server. During this key agreement protocol, it is important to also offer anonymity and unlinkability as otherwise location tracking of a particular device becomes possible and thus might lead to privacy intrusion, especially when the device is linked to a certain user like in WBANs. Moreover, as these devices are mostly battery powered, it is very important to handle the process as efficiently as possible in order to extend the battery life as long as possible. Current proposals in literature on key agreement protocols are based on hash operations, symmetric key, and public key based mechanisms. Hash operations are in general the most efficient [2] and, as we aim for the most efficient approach, we limit our proposed protocols to only hash based constructions and investigate new methods to decrease this number of hash operations. In particular, we exploit the nice feature of the variable output length of the Keccak hash function, which has been standardized in 2015 as the new hash function SHA3. At the moment, this hash function has not the best performance compared to other hash functions in literature, but it has a well proven security strength and the first implementations on chip are already available on the market, resulting in a better overall performance.

We propose two key agreement protocols. In the first protocol, key material is explicitly stored on the server side, resulting in high storage requirements and a lot of overhead for each new registration of device. In addition, during the protocol run, the server needs to look up the particular device into its database in order to find the corresponding key material, which limits the scalability of the system. In the second protocol, there is no storage required on the server side and still the same security features can be obtained, but at some additional communication and computation cost as now the lengths of the transmitted messages are longer and there is one additional hash operation required. In this scheme, the scalability is much larger as now the server only needs to verify if the client belongs to the list of revoked devices, which is typically much shorter compared to the whole set of registered devices. The protocol is proven in the random oracle model, where the hash function is replaced by a random oracle. This type of proof is typically used when no weaker assumptions on the cryptographic hash can be made.

The paper is outlined as follows. In Section 2, we give an overview of relevant related work. Section 3 deals with preliminaries. In Section 4, we present our protocols. The security strength of both protocols, together with a comparison with other related protocols in literature, is discussed in Section 5. The comparison in performance between our proposed protocols and related work is provided in Section 6. Finally, we end the paper with some conclusions in Section 7.

## 2. Related Work

There is a huge amount of papers on authentication and key agreement protocols in a client–server based architecture, both for general settings and application specific domains. In order to limit the related work, we focus on the most recently proposed symmetric key based protocols, consisting of two types of devices able to operate without user interaction. Note that also many schemes exist in literature where password and biometrics related information is required in order to get access to the secret information stored in the device [3,4].

In the domain of smart home automation, the scheme of [5] in 2017 was the first to enable mutual authentication and key agreement between sensors on the one hand and home gateway on the other hand in an anonymous way and without linkability using solely symmetric key based operations. The system is based on asymmetric key distribution between sensors and home gateway in the scheme. The scheme is not resistant against known session temporary specific information because the whole security on the sensor side depends on the knowledge of the random value generated in the beginning of the protocol. It requires the usage of hash functions, symmetric key encryptions, and xor operations.

In the area of Industrial IoT applications, we can distinguish the recent work of [6] in which a lightweight authentication and key distribution (LAKD) scheme has been proposed built of only hash functions, xoring, addition, and subtraction. Their work was inspired on the protocol of [7] in which several weaknesses have been identified by [8,9,10], being a lack of protection against tracking, offline identity guessing, impersonation, and replay attacks. Their scheme [6] consists of four communication phases and a lot of hash calls should be made at the sensor node. It is not clear in their protocol how the gateway remains synchronized with the sensor node and how it can resist synchronization attacks. They show that their scheme outperforms other related work in literature [11,12,13,14] with respect to computational performance. Only [7] is slightly more efficient. In addition, the communication cost of [6] is still lower compared to [12,13,14], but slightly higher than [7,11,14]. However, it has also been clearly shown that all of the schemes [7,11,12,13,14] suffer significant weaknesses with respect to attacks like for instance tracking attack [7,11,13,14], offline identity guessing attack [7,11,12,14], impersonation attack [7,14], denial of service attack [7,11,12,13,14], etc.

In [15], another privacy enabled authentication and key agreement scheme for wearable sensors in wireless body area networks has been proposed, consisting of only xor and hash operations. The scheme is an improvement of [16] in which offline identity guessing, node impersonation, and hub node spoofing attacks have been identified. However, the scheme is not resistant against known session temporary specific information as if the random generated data $r_{n}$ of the tag is known, the identity can be derived of the tag. If the session data of the reader is also retrieved, the whole system is broken. The scheme consists of three hash calls at the side of the sensor. They show that their scheme is more performant with respect to other related schemes in literature [16,17,18,19]. Only the scheme of [20] is more performant but does not offer anonymity and and unlinkability and is not resistant against insider and impersonation attacks. In addition, the scheme of [18] also offers no anonymity and unlinkability. The scheme of [17] suffers from insider attacks.

A recent authentication and key agreement scheme for RFID use cases has been proposed in [21], in which an improvement is made on [22] as it has been shown to not be resistant against collision, denial of service, and stolen verifier attacks. However, their scheme is also not resistant against known session temporary attacks and synchronization attacks. In addition, replay attacks are also possible as the timestamp in the first message is not included in the verification hash of the message and thus can be easily modified and replayed. They compare their scheme with [23,24,25,26] and find that their scheme outperforms the schemes satisfying anonymity [22,25] and are slightly worse than the schemes without anonymity [23,24,26]. Moreover, in all these schemes, several security related problems have been identified.

In order to finally compare our scheme with related work, we will thus focus on the schemes of [5,6,15,21], which represent the latest state-of-the art in the field satisfying the most relevant security features and have already a very good efficiency compared to other work in their respectively application fields.

## 3. Preliminaries

We first discuss the security architecture, attack model, and security features addressed in this scheme. Then, we shortly describe the Keccak hash function and the corresponding security strength that it offers.

### 3.1. Security Architecture

The set-up of the system is very simple and consists solely of three entities, being a service provider, a more constrained client, being typically the sensor and a more powerful server being responsible for the authentication. The service provider plays only a role in the initialization phase of the process. The actual authentication and key agreement is between the last two entities. The server is typically split up in two entities with a secure channel in between. One part is responsible for the handling of the authentication requests and the other part for the storage. To ease the notations and explanations, we consider them here as one entity. Note that we define a generic scheme such that, in our scheme, the client can be any type of sensor or even RFID tag, depending on the use case of smart home, industrial IoT, etc., as mentioned before in related work. From now on, we call it sensor and server, respectively.

### 3.2. Attack Model and Security Features

First of all, we consider the service provider, responsible for the initial sharing of key material between server and sensor, to be completely honest and trustworthy. Moreover, after the initialization process, it removes all generated data out of its memory. Therefore, the initialization process is not included in the security analysis.

#### 3.2.1. Attack Model

The execution of attacks by the adversary is limited to the communication channel between sensor and server. We consider an adversary A that satisfies the following listed characteristics. For each characteristic, the type of attack linked to it is also mentioned.

- The adversary can be a passive or active attacker, and thus is able to listen and collect data on the one hand or actively intercept, modify, or replay data on the other hand. As a consequence, protection should be obtained against replay, impersonation, man-in-the-middle, desynchronization, and denial of service attacks.
- The attacker is able to get access to the session specific variables by means of timing attacks, both on the server and sensor sides. As a consequence, protection must be offered against the known session specific temporary information attack.
- The adversary can capture any sensor and is able to retrieve the data stored on the sensor by means of power analysis techniques. Consequently, perfect forward secrecy is a very important security feature to be addressed since it avoids the attacker to be able to reveal the previously generated session keys.

#### 3.2.2. Assumptions

Note that we do not consider an adversary to get access to the secret key data of the server. This is a reasonable assumption as the server typically is more powerful to be able to offer strong protection. However, in the first protocol with storage, the amount of data to protect is much larger, compared to the second protocol, where the secret key material is only presented by one parameter.

#### 3.2.3. Goals of the Attacker/Security Features

As the ultimate goal of the attacker is to reveal the common shared key generated in the protocol, it is important to construct this key through a mutual authenticated process, where both server and sensor participate in the construction.

The attacker can also be interested in revealing the identity of the sensor in order to provide for instance location tracking of its owner. Therefore, in the latest generation of the schemes, anonymity and unlinkability are two other security features which are often included. In particular, unlinkability avoids activity tracking of its users and is closely related to anonymity.

Furthermore, we also show that our proposed protocols offer perfect forward secrecy and resistance against replay, desynchronization, denial of service, and specific temporary information attacks. Moreover, they do not need synchronized clocks to obtain these features.

### 3.3. SHA3-Keccak

In our scheme, we will limit the usage of operations to xor operations and hashing. For the hash function, we make use of the latest SHA3 standard from 2015 and more in particular the underlying Keccak scheme [27]. SHA3 has been developed in the course of an open call for new hash functions, launched by NIST, due to the fact that the previous variant SHA1 was broken. However, the other previous variant SHA2 is not yet broken, and SHA3 has the main strength that it is open source and the complete design rationale is specified, in contrast to SHA2 which was developed behind closed doors at NSA.

**Figure 1:**
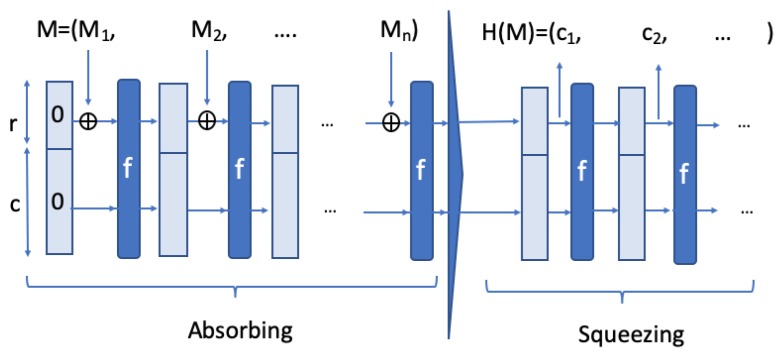
Message M is broken into *n* consecutive *r* bit pieces $M_{1},...,M_{n}$. The output, after applying the permutation *f* several rounds, is denoted by $c_{1},c_{2},...$. The size of the state on which the function *f* works, is called the rate *r*, while the capacity *c* denotes the size of the part that is untouched by input or output [29].

Keccak is based on the interesting concept of sponge construction introduced by its inventors [28]. In this type of construction, any amount of data are first absorbed into the sponge, and then any amount of data are squeezed out without loss in security strength. This last feature in particular will be exploited in our scheme. Figure 1 illustrates the process in the sponge construction.

One of the standardized versions is the SHAKE128(M,d) function on message *M* with variable input size and variable output size *d*. It has been shown that this function has a resistance of at least min$\left(2^{d/2},128\right)$ on collision attacks, preimage, and second preimage attacks. As a consequence, even if the output length is larger than 256, the resistance will still be at least 128 bits. Note that the security strength of a hash function is independent of the input length *M*. For ease of notation, we denote SHAKE128(M,d) as H(M).

## 4. Proposed Protocol

**Figure 2:**
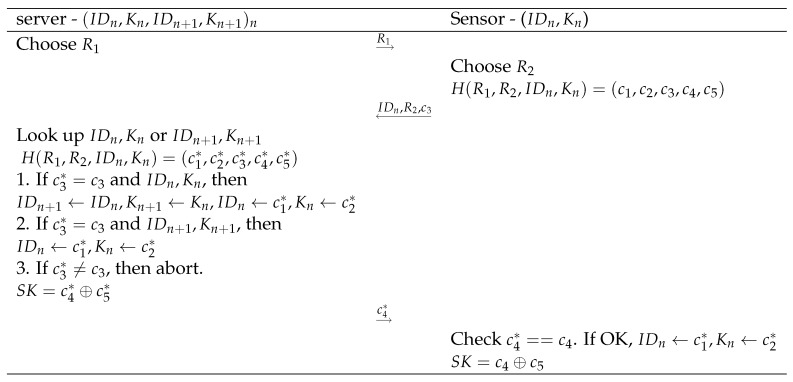
Steps and computations in the proposed authentication and key agreement scheme with storage.

**Figure 3:**
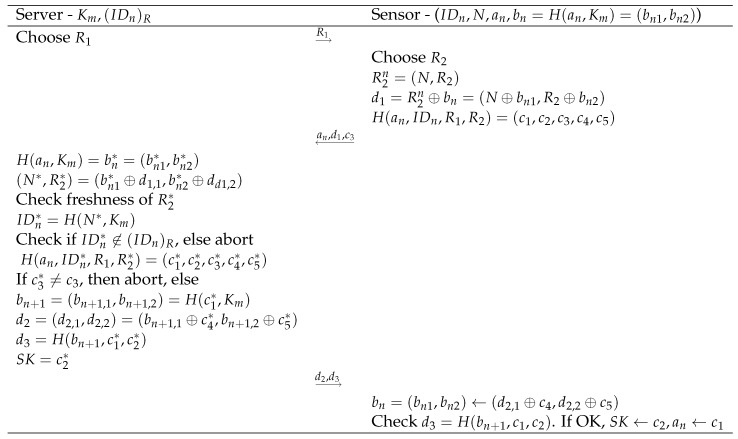
Steps and computations in the proposed authentication and key agreement scheme without storage.

We propose two protocols with similar underlying structure and building blocks. The first one requires permanent storage and update of the key material on the server side, while the second one does not need any storage of individual key material but has a slightly higher communication cost. We distinguish in both protocols an initialization phase and an actual authentication and key agreement phase of the session key SK. Both protocols are also illustrated in Figure 2 and Figure 3.

### 4.1. With Storage

#### 4.1.1. Initialization Phase

In this phase, each sensor receives an identity $ID_{n}$ and a secret key $K_{n}$. These values are stored on the sensor. The index *n* refers to the *n*-th update of identity and key related material. In the database at the server’s side, there are four columns containing the values corresponding with the parameters $ID_{n},K_{n},ID_{n+1},K_{n+1}$. For each new registration of the sensor at the server, the first two columns are completed. The other columns are completed after the first successful authentication and key agreement phase.

#### 4.1.2. Authentication and Key Agreement Phase

To start the process, the sensor sends a triggering request to the server. This is replied by the server with a Hello message, containing a random value $R_{1}$. Note that, in the context of RFID based schemes, the server (reader) starts the communication and this triggering event is not required.

Upon reception of this value, the sensor first derives a new random value $R_{2}$ and computes

$$\begin{array}{ccc} H(R_{1},R_{2},ID_{n},K_{n}) & = & \left(c_{1},c_{2},c_{3},c_{4},c_{5}\right) \end{array}$$

The sensor then stores these values temporary in its memory. Next, it sends $M_{1}=\left\{ID_{n},R_{2},c_{3}\right\}$ to the server.

After receiving $M_{1}$, the server checks the existence of $ID_{n}$ in its database in the columns of $ID_{n}$ or $ID_{n+1}$. If so, it takes the corresponding variable $K_{n}^{*}$, being either $K_{n}$ or $K_{n+1}$ in order to also compute

$$\begin{array}{ccc} H(R_{1},R_{2},ID_{n},K_{n}^{*}) & = & \left(c_{1}^{*},c_{2}^{*},c_{3}^{*},c_{4}^{*},c_{5}^{*}\right) \end{array}$$

There are now three options.

- If $c_{3}^{*}=c_{3}$ and $ID_{n}^{*}=ID_{n}$, the table contents are updated as follows: $ID_{n+1}\leftarrow ID_{n},K_{n+1}\leftarrow K_{n},ID_{n}\leftarrow c_{1}^{*},K_{n}\leftarrow c_{2}^{*}$. The session key is defined as $SK=c_{4}^{*}⊕c_{5}^{*}$. The server sends $c_{4}^{*}$ to the sensor.
- If $c_{3}^{*}=c_{3}$ and $ID_{n}^{*}=ID_{n+1}$, only the following table contents need to be updated $ID_{n}\leftarrow c_{1}^{*},K_{n}\leftarrow c_{2}^{*}$ and $SK=c_{4}^{*}⊕c_{5}^{*}$. The server sends $c_{4}^{*}$ to the sensor.
- If $c_{3}^{*}$ is different from $c_{3}$, the server aborts the process.

When $c_{4}^{*}$ arrives at the sensor, it checks if this value corresponds with the stored value. If so, it also updates its key material $ID_{n}\leftarrow c_{1},K_{n}\leftarrow c_{2}$ and derives the session key as $SK=c_{4}⊕c_{5}$.

### 4.2. Without Storage

#### 4.2.1. Initialization Phase

In this scheme, the server only stores a list of revoked sensors ${\left(ID_{n}\right)}_{R}$ with static identity $ID_{n}$. The server possesses a master key $K_{m}$.

The sensor needs to store the parameters $a_{n},b_{n},ID_{n},N$. The parameter $a_{n}$ represents a random nonce and is unique for each sensor. The parameter $b_{n}=H\left(a_{n},K_{m}\right)=\left(b_{n1},b_{n2}\right)$ is thus split up into two parts. Finally, the fixed identity of each sensor is defined by $ID_{n}=H\left(N,K_{m}\right)$, with *N* a unique nonce and $K_{m}$ the master key of the server.

#### 4.2.2. Authentication and Key Agreement Phase

The protocol starts again with a Hello message from the server containing the random value $R_{1}$ after a triggering request of the sensor.

Next, the sensor chooses its own random value $R_{2}$ and defines $R_{2}^{n}=\left(N,R_{2}\right)$. It computes $d_{1}=\left(d_{1,1},d_{1,2}\right)=R_{2}^{n}⊕b_{n}=\left(N⊕b_{n1},R_{2}⊕b_{n2}\right)$ and

$$\begin{array}{ccc} H(a_{n},ID_{n},R_{1},R_{2}) & = & \left(c_{1},c_{2},c_{3},c_{4},c_{5}\right) \end{array}$$

The values $\left(c_{1},c_{2},c_{3},c_{4},c_{5}\right)$ are stored in the sensor before sending $M_{1}=\left\{a_{n},d_{1},c_{3}\right\}$ to the server.

The server first defines $H\left(a_{n},K_{m}\right)=b_{n}^{*}$ and derives from $d_{1}$ both $N^{*},R_{2}^{*}$, resulting also in $ID_{n}^{*}=H\left(N^{*},K_{m}\right)$. Then, the server checks the freshness of $R_{2}^{*}$. For instance, suppose that it uses in a given interval the same $R_{1}$, then all received values $R_{2}^{*}$ should be fresh. Next, if $ID_{n}^{*}$ does not belong to the list of revoked sensors, the process continues; otherwise, the protocol stops. Next, the server can also compute

$$\begin{array}{ccc} H(a_{n},ID_{n}^{*},R_{1},R_{2}^{*}) & = & \left(c_{1}^{*},c_{2}^{*},c_{3}^{*},c_{4}^{*},c_{5}^{*}\right) \end{array}$$

There are now two possibilities. In case $c_{3}\neq c_{3}^{*}$, the server aborts the process. Otherwise, the server defines

$$\begin{array}{ccc} SK & = & c_{2}^{*}\\ b_{n+1} & = & \left(b_{n+1,1},b_{n+1,2}\right)=H\left(c_{1}^{*},K_{m}\right)\\ d_{2} & = & \left(d_{2,1},d_{2,2}\right)=\left(b_{n+1,1}⊕c_{4}^{*},b_{n+1,2}⊕c_{5}^{*}\right)\\ d_{3} & = & H(b_{n+1},c_{1}^{*},c_{2}^{*}) \end{array}$$

The message $M_{2}=\left\{d_{2},d_{3}\right\}$ is sent to the sensor.

Finally, the sensor derives $b_{n}\leftarrow \left(d_{2,1}⊕c_{4}^{*},d_{2,2}⊕c_{5}^{*}\right)$ using the stored values $\left(c_{1},c_{2},c_{3},c_{4},c_{5}\right)$. Next, it checks the validity of $d_{3}$ by computing $H(b_{n+1},c_{1}^{*},c_{2}^{*})$. If not correct, it aborts the process. Otherwise, the variable $a_{n}\leftarrow c_{1}$ is updated and the common shared secret key $SK=c_{2}$.

Note that we kept the same flow for both protocols in order to be able to easily switch from one to another and to use the same type of proof structure. It is very easy to reshape the protocol into a 2-phase protocol using similar ideas.

## 5. Security Evaluation

First of all, we give a formal proof of the protocol under the random oracle model for the adversary A defined in Section 3. Based on this proof, we discuss the design choice of the lengths of the parameters in order to obtain a security strength of 128 bits. Then, we define the strength of both protocols in an informal way with respect to the main security attacks and security features. Finally, we compare our protocol from a security point of view with respect to other related and recent protocols as mentioned in Section 2.

### 5.1. Security Strength and Concrete Parameters

Let us denote the minimum output length of the hash function by $n_{d}$ and the minimum output length of a variable by $n_{v}$. We now prove the security of our proposed protocols in the random oracle model, following the method described in [30] and applied in e.g., [31] with an adversary A as defined in Section 3. We focus on the actual key agreement and not on the initialization phase, as we consider the service provider to be a fully trusted entity.

The participants *U* in the scheme are the Sensor T and server R on the one hand and a random oracle *O* on the other hand, i.e., U={R,T,O}. Taking into account the properties of the adversary, we assume that the attacker can run the following queries:

- Hash queries H(m). If *m* already exists in the list $L_{H}$, the value H(m) will be returned. Otherwise, a random value will be generated, added to the list $L_{H}$, and returned.
- Send queries. These queries simulate active attacks, where an adversary can modify the transmitted messages. As a result, a corresponding reply will be generated:
  - Send(0,R). First, a random variable $R_{1}$ is chosen and sent to the sensor.
  - Send($R_{1}$,T). In addition, a random variable $R_{2}$ is chosen. A message $M_{1}$ containing the outcome of the hash function on the random data, identity and key related data and also information to identify the sensor and corresponding key material is generated and sent to the server. The outcome of the hash function is stored by the sensor.
  - Send($M_{1}$,R). The correctness of the message $M_{1}$ can be verified by checking the outcome of the hash value. For that, the server should look up or derive the identity related data of the sensor and the corresponding key material. If correct, the session key is defined. In the protocol without storage, also new identity related data are defined and masked in order to send it to the sensor. Finally, message $M_{2}$, based also on the output of the hash function derived in the previous step, is sent to the sensor.
  - Send($M_{2}$,T). The received values of $M_{2}$ are checked for correctness using the stored values of the hash function. If correct, the session key is also computed and the key agreement protocol has been successfully ended.
- Execute queries. These queries simulate the passive attacks, where an adversary can eavesdrop onto the channel and collect the transmitted messages. There are three different execute queries resulting from the first three send queries defined above.
- Session specific state reveal queries (SSReveal). According to the adversary model of A, the attacker is able to retrieve session specific state information, derived by the T and R, respectively. For the protocol with storage, no more information already available from the execute queries, can be found in this way. For the protocol without storage, $R_{2}$ is revealed, since it is hidden in the variable $d_{1}$ of $M_{1}$.
- Corrupt queries. These queries give the secret key material of the entity as result and need to be added to prove the perfect forward security feature. Note that only Corrupt (T) is defined in the adversary model A. As the service provider is considered to be a trusted entity and not included in the security model, there are no corrupt queries with regard to the service provider.
- Session key reveal query (SKReveal). In this query, the established symmetric SK between T,R is returned if it has been successfully generated.
- Test query. In this query, either the established SK or a random value is returned, dependent on the output c=1 or c=0 respectively of a flipped coin *c*. Note that the test query cannot be issued when SKReveal or corrupt queries have been executed.

In order to prove the semantic security of the scheme, we consider the following two definitions:

- The entities T and R are partners if they are able to successfully derive in a mutual authenticated way a common shared SK.
- The established shared secret key is said to be fresh if the SK has been established without SKReveal queries by the adversary or Corrupt queries of T.

The final goal of the adversary A is to successfully predict the outcome of the test query, defined above. Consequently, a successful attacker is able to distinguish the difference between a real secret session key and a random value. The advantage of the adversary A in breaking the semantic security of the proposed scheme equals to Adv(A)=|2Pr[succ]−1|, with Pr(succ) being the probability that the adversary has success. As a result, we can say that the proposed protocols offer semantic security under the random oracle model for adversaries A if Adv(A)≤ϵ, for any sufficiently small ϵ >0.

In the proof below, we make use of the following difference lemma, defined in [32].

**Lemma** **1.** *(Difference Lemma) Let $E_{1},E_{2}$ be the events of winning Game 1 and Game 2. Denote an error event by E, such that $E_{1}|\neg E$ occurs if and only if $E_{2}|\neg E$. Then, $|Pr\left[E_{1}\right]-Pr\left[E_{2}\right]|\leq Pr\left[E\right]$.*

**Theorem** **1.** *Let A be a polynomial time adversary against the semantic security, which makes a maximum of $q_{s}$ Send queries, $q_{e}$ Execute queries and $q_{h}$ Hash queries. The advantage of A is bounded by $Adv\left(A\right)\leq \frac{O{\left(q_{s}+q_{e}\right)}^{2}}{2^{n_{v}+1}}+\frac{O{\left(q_{h}\right)}^{2}}{2^{n_{d}}}+\frac{O{\left(q_{s}\right)}^{2}}{2^{n_{d}+1}}$.*

**Proof.**  We proof the theorem by means of game hopping [32] and by applying Lemma 1. As a consequence, we exploit the fact that an attacker’s success probability only increases by a negligible amount when moving between the games. Five games {GM0,GM1,GM2,GM3,GM4} are defined and their corresponding probability of the attacker winning the game is denoted by $succ_{i}$ for 0≤i≤4.

- Game GM0. This is the original and real game defined in the semantic security framework and is defined as
  (1) $$\begin{array}{c} Adv\left(A\right)=|2Pr\left[succ_{0}\right]-1|. \end{array}$$
- Game GM1. In GM1, the random oracle simulates the different queries and outputs the corresponding results in the lists. Following the definition of random oracle model, we have that
  (2) $$\begin{array}{c} Pr\left[succ_{1}\right]=Pr\left[succ_{0}\right]. \end{array}$$
- Game GM2. In this game, all oracles are also simulated, but now with avoiding collisions in the output of the hash function and the selection of random values $R_{1},R_{2}$ among the different sessions. Consequently, due to the difference lemma and birthday attacks, it holds that
  (3) $$\begin{array}{c} |Pr\left[Succ_{2}\right]-Pr\left[Succ_{1}\right]|\leq \\ \frac{O{\left(q_{s}+q_{e}\right)}^{2}}{2^{n_{v}+1}}+\frac{O{\left(q_{h}\right)}^{2}}{2^{n_{d}+1}}. \end{array}$$
- Game GM3. In this game, the adversary A is able to find the hash value ($c_{3}^{*}$ or $d_{3}$ for protocol with and without storage respectively) without input of the random oracle Send queries. In this case, the scheme is simply stopped. Consequently, GM2 and GM3 are indistinguishable, except when the T rejects this value. Thus, by applying the difference lemma, we have that
  (4) $$\begin{array}{c} |Pr\left[Succ_{3}\right]-Pr\left[Succ_{2}\right]|\leq \frac{O{\left(q_{s}\right)}^{2}}{2^{n_{d}+1}}. \end{array}$$
- Game GM4. In this game, we consider the specific adversary model in which either the session state variables (corresponding to SKReveal query) can be revealed or the secret variables (corresponding to Corrupt query) at T. The adversary can perform Execute and Hash queries in order to find the SK:
  - In the case of SKReveal(T) and SKReveal(R), the SK can still not be retrieved if the hash function is secure for collision and preimage attacks as it is constructed based on the output of the hash function, which includes both session state variables and secret key variables of both entities as input.
  - If Corrupt(T) is applied, the previously generated session keys cannot be retrieved as they require the secret key information, which are the input of a hash function with the new key material as output. Again, the usage of a hash function resistant for collision and preimage attacks avoids the success of this attack.
  Consequently, the difference between GM3 and GM4 is negligible as long as the hash function is secure for collision and preimage attacks. Therefore,
  (5) $$\begin{array}{c} |Pr\left[Succ_{4}\right]-Pr\left[Succ_{3}\right]|\leq \frac{O{\left(q_{h}\right)}^{2}}{2^{n_{d}+1}}. \end{array}$$

Finally, applying Lemma 1 on the games GM0, GM1, GM2, GM3, and GM4, taking into account Equations (1), (2), (3), and (5), results in the final proof of the theorem. □

**Theorem** **2.** *In order to obtain 128 bits of security in the protocol without storage, $|ID_{n}\left|,\right|K_{n}\left|,\right|R_{1}\left|,\right|R_{2}\left|,|SK\right|\geq 128$ bits and $|c_{3}|\geq 256$ bits.*

**Proof.**  From Theorem 1, we should avoid collisions in the hash output and thus $|c_{3}|\geq 256$ bits. As also collisions in the random variables and resistance against exhaustive search should be offered, it follows that $|ID_{n}\left|,\right|K_{n}\left|,\right|R_{1}\left|,\right|R_{2}|\geq 128$ bits. □

**Theorem** **3.** *In order to obtain 128-bit of security in the protocol with storage, $|K_{m}\left|,\right|R_{1}\left|,\right|R_{2}\left|,|SK|,|N|,\right|b_{n2}\left|,\right|d_{2,2}|\geq 128$ bits and $|ID_{n}\left|,\right|b_{n}\left|,\right|d_{1}\left|,\right|c_{3}\left|,\right|d_{2}\left|,\right|d_{3}\left|,\right|b_{n1}\left|,\right|d_{2,1}|\geq 256$ bits.*

**Proof.**  From Theorem 1, we should avoid collisions in the hash output and thus clarifies the restrictions on the sizes of $ID_{n},d_{1},c_{3},d_{3}$. Since we also need to protect against the session reveal attacks, it follows that $b_{n1},d_{2,1}$ should also both have the same restriction. □

The size of the other variables $K_{m},R_{1},R_{2},N,SK,b_{n2},d_{2,2}$ are chosen in such a way that they avoid collisions and exhaustive search and thus clarify the minimum size of 128 bits.

### 5.2. Informal Security Analysis

Table 1 summarizes the security strength for both protocols with respect to the most important and relevant security features and attacks.

The protection against replay attacks, session specific temporary information attacks and denial of service attacks is similar as both protocols rely on the same building blocks and structure. In addition, in order to obtain mutual authentication, the strength of the Keccak protocol is exploited in the two protocols.

Dynamic identities of the sensor are used in both protocols to obtain anonymity and unlinkability. However, the process how to construct these identities is completely different in the two protocols. In the protocol with storage, a synchronized version needs to be stored and updated at the server side, and the sensor is able to update its key material independent of additional input of the server. In the protocol without storage, the identity of the sensor should be updated using additional data of the server as its master key is involved in the construction. Consequently, protection against desynchronization attacks is different for the two protocols. While this protection naturally follows from the derivation of the key material in the protocol without storage, it requires additional storage of the previous identity and key of the sensor at the server for the protocol with storage.

Both protocols offer perfect forward secrecy of the sensor, but not for the server. For the protocol without storage, only one parameter needs to be perfectly protected in order to avoid this attack. If this master key is revealed, all previous session keys and identities can be retrieved. In the protocol with storage, the devastating consequences are less as only at most two last session keys per sensor can be revealed, but it also requires the protection of the whole database containing this identity and secret key material of the sensors.

### 5.3. Comparison of Security Features of Different Protocols

Table 2 compares the two proposed security protocols, with and without storage on the server side, with the schemes proposed in [5,6,15,21]. Therefore, we take into account the different security features mentioned in Table 1. Consequently, from Table 2, we can conclude that our proposed protocols are able to offer most of the required security features. The only feature that is not addressed is the perfect forward secrecy on the server side. The other schemes in literature without storage [5,15] also do not satisfy this feature. However, in the schemes where the feature is addressed [6,21], there is no protection against synchronization attacks. Note that recently, in [33], a symmetric key based authentication scheme has been proposed which satisfies at the same time complete perfect forward secrecy and a solution for potential desynchronization problems. However, this scheme does not provide anonymity and consists of five phases with multiple hash functions.

In addition, we want to explicitly note that, for both protocols, there is no need for synchronized clocks on both sensor and server side in order to resist for replay or synchronization attacks. This is typically a very difficult requirement to realize in practice and in particular when constrained devices are involved.

## 6. Performance

In this section, we will discuss the efficiency of our protocol with respect to both computational and communicational costs and compare again with the related work of [5,6,15,21].

For the computational complexity, we restrict the analysis to the most constrained device participating in the authentication and key agreement protocol, being the sensor. We also neglect xor operations and other low cost operations like concatenations, comparisons, splitting, etc, since their impact is negligible compared to the cost of a hash function and encryption operation. In all protocols in order to achieve mutual authentication, one random value should be generated on the sensor side. Therefore, we do not explicitly included this operation in Table 3, summarizing the comparison of the performance of the different protocols. As a consequence, only the amount of hashes and encryptions/decryptions is taken into account. From Table 3, we can conclude that our two proposed protocols have the lowest amount of hash functions and thus the lowest computational complexity, compared to state of the art related work. Only [21] has the same amount as hash functions than our protocol without storage, but this protocol only offers limited security strength as shown in Table 2.

To give an idea of the feasibility and performance of such hash operation on a device, we consider the performance results of [34], where the efficiency of both SHA2 and SHA3 is analyzed on the MAXREFDES♯100 health sensor platform [35], which uses the MAX32620 96 MHz ARM Cortex-M4F microcontroller consisting of 2 MB flash and 256 KB RAM. They measured a timing of 108 μs and 438 μs for one SHA2 and SHA3 call, respectively. Note that the maximum input size of the algorithms is equal to 55B for SHA2 and 135B for SHA3. Taking these numbers into account, Table 3 shows that, in case of SHA2 implementation, Refs. [5,15] become more efficient than our protocol without storage using SHA3. On the other hand, our protocol with storage is still the most efficient one. However, note that researchers are actively working on the construction of more efficient sponge functions in hash functions. Moreover, if SHA3 dedicated chips are used, these numbers would also drastically change and thus it shows the importance of decreasing the number of hash operations to the maximum.

In order to compare the communicational complexity of our protocols with [5,6,15,21], we also assume 128 bit security in these protocols thus resulting in output hashes of 256 bits in length. In addition, we consider timestamps with length equal to 32 bits. Table 4 shows that our protocol with storage on the server side behaves most optimally and has the lowest amount of sent and received bits from the sensor. The protocol without storage behaves a bit worse than [5,21], but, as can be seen from Table 1, offers also more security features.

## 7. Conclusions

In this paper, we have proposed two authentication and key agreement protocols to be applied in any type of client–server architecture, which is in particular very interesting in case the client is resource constrained. The protocols exploit the variable output length of the Keccak algorithm. The security of the protocol is explicitly proven in the random oracle model, which also allows for deriving the sizes of the different parameters used in the scheme. We have shown that our protocols outperform the state of the art with respect to computational cost by requiring only one execution of the hash function on the client side for the protocol with storage and two hash functions for the protocol without storage. In addition, the communication cost is low compared to related work and takes into account the available security features. It is still an open question how to include the perfect forward secrecy from the server side in an efficient way into the schemes.

## Figures and Tables

**Table 1:** Informal security analysis of both protocols.

Characteristic	Protocol with Storage	Protocol without Storage
Mutual authentication	Only the entities knowing the secret key $K_{n}$ or $K_{n+1}$ can derive the correct authentication values $c_{3}$ and $c_{4}$ respectively. Note that thanks to this feature, also protection against impersonation and man-in-the-middle attacks is offered.	The server is the only entity, who is able to derive $b_{n}$ and thus $R_{2}$ from the received value $a_{n}$. The sensor is ensured about the authentication of the server by checking the correctness of $d_{3}$, which cannot be manipulated without knowledge of $K_{m}$. Consequently, the protocol is also resistant for impersonation and man-in-the-middle attacks.
Anonymity and unlinkability	The identity $ID_{n}$ sent in the protocol is dynamic and not linked to a certain static sensor. Its content changes after each successful run of the protocol and therefore no tracking of a specific sensor can be obtained. In addition, the other parameters sent in the protocol have no link with identity related information.	The identity related information sent in the protocol, $a_{n},d_{1}$ is dynamic and updated after each successful run of the protocol. Without knowledge of the secret key $K_{m}$, the attacker is not able to reveal the real identity of the sensor. Moreover, as there is no relation between consecutive parameters $a_{n},d_{1}$, an attacker cannot perform location tracking attacks.
Perfect forward secrecy of sensor.	If an attacker captures the sensor and gets access to $\left(ID_{n},K_{n}\right)$, it will not be able to generate the previous session keys as they were built using the hash function of the previously secure keys $K_{n-1}$, which are overwritten in memory with the current version $K_{n}$. Note that perfect forward secrecy does not hold at the server side. If an attacker gets access to the database, it is able to generate using the collected random values sent in clear in the transmission channel the last established session keys.	If the secret information $a_{n},b_{n},ID_{n},N$ is leaked from the sensor, the previous session keys cannot be revealed as they require the knowledge of the previous $b_{n}$ and also $R_{2}$ values. Without knowledge of $K_{m}$, this data cannot be revealed from the transmitted messages. In addition, the anonymity and unlinkability features will still be valid as the parameters $a_{n},d_{1}$ change after each successful authentication. In addition, here, no perfect forward secrecy on the server side is obtained because if $K_{m}$ is retrieved, the values $b_{n},ID_{n}$ can be derived from the message $M_{1}$ sent by the sensor, resulting in the derivation of SK.
Replay attacks	The value $R_{1}$ can be replayed, but randomness will still be guaranteed by the generation of the random value $R_{2}$ by the sensor. Moreover, as the parameters $\left(ID_{n},K_{n}\right)$ change after each protocol run and a synchronized version is kept at the database, replay attacks are avoided.	In addition, here, $R_{1}$ can be replayed, but randomness will still be guaranteed by the generation of the random value $R_{2}$ by the sensor, whose uniqueness is specifically tested by the server. As a consequence, an attacker cannot obtain two times the same outcome of the hash value.
Desynchronization attack	Suppose the message $M_{1}$ is dropped by the attacker. In this case, both server and sensor are not updated. However, in case the last message $c_{4}^{*}$ is dropped, the server gets updated and not the sensor. Therefore, in order to overcome potential desynchronization in the next call of the protocol, we need to store always the previous values of identity and key material too at the side of the server, which is considered to be the most powerful device.	Due to the nature of the protocol by the specific construction of the key material, there is no synchronization required. In particular, the usage of the static master key $K_{M}$ will always lead to common shared key material.
Denial of service attack	The only place where a potential denial of service attack can appear is in the first step of sending the random value $R_{1}$. However, the sensor can built in a mechanism to block in case more than a threshold of invalid responses are sent back. All the other messages can be specifically verified for the correctness as they include checks on the existence of the key material. Consequently, as the protocol only consists of three phases, the server can never be blocked by having too many open sessions.	The same reasoning for protection against the denial of service attack also holds in this protocol.
Session specific temporary information	In this protocol, there is no additional session specific temporary information that can be revealed in order to be exploited for the generation of the SK.	If also $R_{2}$ is leaked, $b_{n_{2}}$ can be retrieved and thus a collision attack on $b_{n1}$ can be executed. However, the size of $b_{n1}$ is chosen in such a way that it still offers 128-bit protection.

**Table 2:** Comparison of security strength with related and recent literature with respect to the following features: F1: Mutual authentication, F2: Anonymity and unlinkability, F3: Perfect forward secrecy, F4: Resistance against replay attacks, F5: Resistance against desynchronization attacks, F6: Resistance against denial of service attacks, F7: Resistance against specific temporary information, F8: No need for synchronized clocks (Y: Yes, N: No).

Scheme (Authors+Year)	F1	F2	F3	F4	F5	F6	F7	F8
Kumar et al., 2017 [5]	Y	Y	Y	Y(T)	Y	Y	N	N
Chen et al., 2018 [15]	Y	Y	Y	Y(T)	Y	Y	N	N
Mansoor et al., 2019 [21]	Y	Y	Y	N(T,R)	N	N	N	N
Lara et al., 2020 [6]	Y	Y	Y	Y(T,R)	N	Y	Y	N
With storage	Y	Y	Y	Y(T)	Y	Y	Y	Y
Without storage	Y	Y	Y	Y(T)	Y	Y	Y	Y

**Table 3:** Comparison of computational cost with related and recent literature. $N_{H}$ equals the number of hashes and $N_{e}$ the number of encryption operations.

Scheme (Authors+Year)	Nr of Operations	With SHA2 (μs)	With SHA3 (μs)
Kumar et al., 2017 [5]	$2N_{H}+2N_{E}$	564	1116
Chen et al., 2018 [15]	$5N_{H}$	1080	2190
Mansoor et al., 2019 [21]	$2N_{H}$	540	786
Lara et al., 2020 [6]	$9N_{H}$	1944	3942
With storage	$1N_{H}$	-	438
Without storage	$2N_{H}$	-	876

**Table 4:** Comparison of communication cost with related and recent literature.

Scheme (Authors+Year)	Nr of Sent Bits	Nr of Received Bits	Total Sent+Received Bits
Kumar et al., 2017 [5]	704	416	1120
Chen et al., 2018 [15]	1056	1024	2080
Mansoor et al., 2019 [21]	672	416	1088
Lara et al., 2020 [6]	1088	1088	2176
With storage	512	384	896
Without storage	786	640	1426

## References

[1] Lee I., Lee K. (2015). The Internet of Things (IoT): Applications, investments, and challenges for enterprises. Bus. Horiz..

[2] Shou L., Li X., Yeh K.H., Su C., Chiu W. (2019). Lightweight IoT based authentication scheme in cloud computing circumstance. Future Gener. Comp. Syst..

[3] Sudhakar T., Natarajan V. (2019). A new three-factor authentication and key agreement protocol for multi-server environment. Wirel. Netw..

[4] Braeken A. (2015). Efficient anonym smart card based authentication scheme for multi-server architecture. Int. J. Smart Home.

[5] Kumar P., Braeken A., Gurtov A., Iinatti J., Ha P.H. (2017). Anonymous secure framework in connected smart home environments. IEEE Trans. Inf. Forensics Secur..

[6] Lara E., Aguilar L., Sanchez M.A., García J.A. (2020). Lightweight Authentication Protocol for M2M Communications of Resource-Constrained Devices in Industrial Internet of Things. Sensors.

[7] Esfahani A., Mantas G., Matischek R., Saghezchi F.B., Rodriguez J., Bicaku A., Maksuti S., Tauber M.G., Schmittner C., Bastos J. (2019). Lightweight Authentication Mechanism for M2M Communications in Industrial IoT Environment. IEEE Internet Things.

[8] Aghili S.F., Mala H. (2018). Breaking a Lightweight M2M Authentication Protocol for Communications in IIoT Environment. IACR Cryptol. ePrint Arch..

[9] Limbasiya T., Soni M., Mishra S.K. (2018). Advanced formal authentication protocol using smart cards for network applicants. Comput. Electr. Eng..

[10] Adeel A., Ali M., Khan A.N., Khalid T., Rehman F., Jararweh Y., Shuja J. (2019). A multi-attack resilient lightweight IoT authentication scheme. Trans. Emerg. Telecommun. Technol..

[11] Han J., Kim J. A lightweight authentication mechanism between IoT devices. Proceedings of the 2017 International Conference on Information and Communication Technology Convergence (ICTC).

[12] Qiu Y., Ma M. An authentication and key establishment scheme to enhance security for M2M in 6LoWPANs. Proceedings of the 2015 IEEE International Conference on Communication Workshop (ICCW).

[13] Renuka K., Kumari S., Zhao D., Li L. (2019). Design of a Secure Password-Based Authentication Scheme for M2M Networks in IoT Enabled Cyber-Physical Systems. IEEE Access.

[14] Joshitta R.S.M., Arockiam L. Device authentication mechanism for IoT enabled healthcare system. Proceedings of the 2017 International Conference on Algorithms, Methodology, Models and Applications in Emerging Technologies (ICAMMAET).

[15] Chen C.M., Xiang B., Wu T.Y., Wang K.H. (2018). An Anonymous Mutual Authenticated Key Agreement Scheme for Wearable Sensors in Wireless Body Area Networks. Appl. Sci..

[16] Chen J., Gui Z., Ji S., Tan H., Tang Y. (2018). Cloud-aided lightweight certificateless authentication protocol with anonymity for wireless body area networks. J. Netw. Comput. Appl..

[17] Liu J., Zhang Z., Chen X., Kwak K.S. (2014). Certificateless Remote Anonymous Authentication Schemes for WirelessBody Area Networks. IEEE Trans. Parallel Distrib. Syst..

[18] Zhao Z. (2014). An Efficient Anonymous Authentication Scheme for Wireless Body Area Networks Using Elliptic Curve Cryptosystem. J. Med. Syst..

[19] Abbasinezhad-Mood D., Nikooghadam M. (2018). Efficient Anonymous Password-Authenticated Key Exchange Protocol to Read Isolated Smart Meters by Utilization of Extended Chebyshev Chaotic Maps. IEEE Trans. Ind. Inform..

[20] Li X., Ibrahim M.H., Kumari S., Sangaiah A.K., Gupta V., Choo K.K.R. (2017). Anonymous Mutual Authentication and Key Agreement Scheme for Wearable Sensors in Wireless Body Area Networks. Comput. Netw..

[21] Mansoor K., Ghani A., Chaudhry S.A., Shamshirband S., Ghayyur S.A.K., Mosavi A. (2019). Securing IoT-Based RFID Systems: A Robust Authentication Protocol Using Symmetric Cryptography. Sensors.

[22] Gope P., Hwang T. (2015). A realistic lightweight authentication protocol preserving strong anonymity for securing RFID system. Comput. Secur..

[23] Yang J., Park J., Lee H., Ren K., Kim K. Mutual authentication protocol. Proceedings of the Workshop on RFID and Lightweight Crypto.

[24] Tan C.C., Sheng B., Li A. (2008). Secure and serverless RFID authentication and search protocols. IEEE Trans. Wirel. Commun..

[25] Cai S., Li Y., Li T., Deng R.H. Attacks and improvements to an RIFD mutual authentication protocol and its extensions. Proceedings of the Second ACM Conference on Wireless Network Security.

[26] Cho J.S., Jeong Y.S., Park S.O. (2015). Consideration on the brute-force attack cost and retrieval cost: A hash-based radio-frequency identification (RFID) tag mutual authentication protocol. Comput. Math. Appl..

[27] Bertoni G., Daemen J., Peeters M., Van Assche G. (2017). The Keccak SHA-3 submission. SHA-3 Competition.

[28] Bertoni G., Daemen J., Peeters M., van Assche G. Sponge Functions. https://pdfs.semanticscholar.org/0338/0dd678b5dbf37734452ac57f793db1a9620c.pdf.

[29] SHA3 Wikipedia, Retrieved 10-3-2020. https://en.wikipedia.org/wiki/SHA-3.

[30] Pointcheval D., Zimmer S. (2008). Multi-factor authenticated key exchange. Appl. Cryptogr. Netw. Secur..

[31] Braeken A., Kumar P., Martin A. (2018). Efficient and Provably Secure Key Agreement for Modern Smart Metering Communications. Energies.

[32] Shoup V. (2004). Sequences of Games: A Tool for Taming Complexity in Security Proofs. http://eprint.iacr.org/2004/332/.

[33] Avoine G., Canard S., Ferreira L. (2019). Symmetric-key authenticated key exchange (SAKE) with perfect forward secrecy. Cryptol. ePrint Arch..

[34] Winderickx J. (2020). Energy-Efficient and Secure Implementations for the IoT. Ph.D. Thesis.

[35] MAXIM Integrated, MAXREFDES100♯: Health Sensor Platform. https://www.maximintegrated.com/en/design/reference-design-center/system-board/6312.html.

